# Development of a Safety Toolkit to Influence Inclusion Barriers for Adolescents and Young Adults (AYA) in Adult Clinical Trials

**DOI:** 10.1007/s43441-025-00761-7

**Published:** 2025-03-13

**Authors:** Devona Williams, Julie Maidment, Pamela Concepcion, Gyorgy Zorenyi

**Affiliations:** Global Patient Safety, Oncology, Astrazeneca, 101 Orchard Ridge Dr., Gaithersburg, MD 20878 USA

**Keywords:** Benefit-risk, Pharmacovigilance, AYA, Cancer research, Protocol, Risk assessment

## Abstract

Lack of long-term safety data for the AYA population has been identified as a key area that reduces enrolment of AYA in adult oncology clinical trials. Here we describe a potential safety assessment solution, from a pharmacovigilance and clinical patient safety perspective, to enhance the inclusion of adolescents into adult oncology trials. To help bridge gaps in safety data that limit AYA participation, a Patient Safety Oncology Toolkit for AYA patients has been developed. The safety toolkit includes recommended additional clinical study protocol templated wording for assessment and management of general AYA-related risks for oncology agents, including infertility, growth and development, new primary malignancies, and neurocognitive effects. There is also recommended language to incorporate into the study protocol for investigational product specific risk considerations based on impacted organ systems. Using the safety toolkit, a key deliverable from the evaluation of the risks is the generation of a safety go, or no-go, Red-Amber-Green (RAG) rating for each study. The RAG rating scale is intended to summarize the scope and severity of any specific treatment-related safety concerns and helps standardize company governance and investment decisions. This toolkit is intended to allow teams to safely include AYA individuals in adult oncology studies and allow this population better access to life-changing medicines.

## Introduction

Categorization of cancer patients by age group is typically stratified into one of two larger groups: pediatric or adult. Maintaining this narrowed view of age groups, however, neglects a population that requires specific consideration. Adolescent and young adult (AYA) patients, as defined by professional societies such as National Cancer Institute (NCI) European Society of Medical Oncology (ESMO) and National Comprehensive Cancer Network (NCCN) describes a distinct population of subjects aged 15–39. For the purposes of this publication, the risk analysis methods described will consider unique key safety risks for patients ages 12–18. AYA patients face unique treatment challenges in cancer care that impact optimal treatment selection, survival outcomes and long-term safety. In line with these unique challenges, the AYA cancer population have lower rates of enrolment in clinical trials compared to adult cancer patients [1].

AYA patients have distinctive elements associated with their care, including biologic differences, socioeconomical concerns, survivorship challenges and psychosocial issues, that impact treatment efficacy and cancer outcomes [2, 3]. Cancer outcomes for the AYA population are worse than those reported for pediatric or adult patients [4, 5]. Limitations in protocol selection, treatment setting and inclusion in clinical trials have been shown to have a negative impact on survival and clinical response in AYA patients [6, 7]. Lack of clinical trial enrolment and specialised physician experience with this age group are contributors toward knowledge gaps that exist in treatment of AYA cancer patients. Better understanding of the specific benefits/risks and therapy tolerability for this population will play an important role in providing increased opportunities for enrolment and accrual of AYA subjects in clinical trials. Increased access to clinical trials holds great potential to close these knowledge gaps and progress towards optimal outcomes for AYA patients [8]. A systematic literature review identified several barriers and facilitators to enrolment of AYA in cancer trials. Barriers identified included insufficient staff and resources, lacking physician expertise and education, limited trial availability, restrictive eligibility criteria and lack of communication between pediatric and adult oncology teams [7, 9].

The pharmaceutical industry can play a key role in expanding access to clinical trials for investigational cancer therapies via modification of clinical trial eligibility criteria. During the drug development process, companies must recognize and explore the biologic differences in tumour genomic histotypes and molecular features that may differ in AYA subjects, to develop effective therapies. Additionally, in the era of personalized cancer medication, if the focus is on molecular targets instead of disease indications, that may expand the available number of clinical trials open to AYA patients. It is also important for companies that direct research and development of new medicines to develop accurate measures to account for and to study the differences in the physiology of AYA patients that may impact the pharmacokinetics and pharmacodynamics of investigational medicinal agents [10].

This safety toolkit is intended to lessen the barriers of trial availability and impact education limitations in physician and trial site staff surrounding safety considerations for AYA subjects in cancer trials. Clinical trial sponsors will be empowered to proactively assess safety risks and create mitigation techniques, increasing clinical trial options open to these patients. This will also help to augment the education of site physicians and staff, to enhance strategies that improve treatment outcomes by predicting, managing, and supporting therapy complications, in an ultimate effort to optimize therapy outcomes.

At AstraZeneca, we are committed to the concepts of diversity and patient centricity in how we plan and conduct clinical trials. As a part of these commitments, our future aim is to explore the inclusion of all AYA patients (age of enrolment lowered to 15, or if appropriate to age 12) in adult oncology clinical trials, including early phase studies, where there is sound scientific rationale and an unmet need within the AYA population. To support these goals, we have developed a safety toolkit to enable teams to assess medication safety risks for the AYA population in a structured way, allowing product teams to consider lowering the age of inclusion in adult oncology trials. The toolkit includes a decision tree that allows teams to determine whether safety risks and tolerability are significantly different for AYAs compared to adults (i.e., if the risk is more severe, impacts different organ systems or occurs with an increased frequency), and then whether additional protocol changes, i.e. AYA-specific safety monitoring or mitigation is required. AYA- specific clinical protocol template language is provided as an accompaniment to the toolkit.

**Figure 1:**
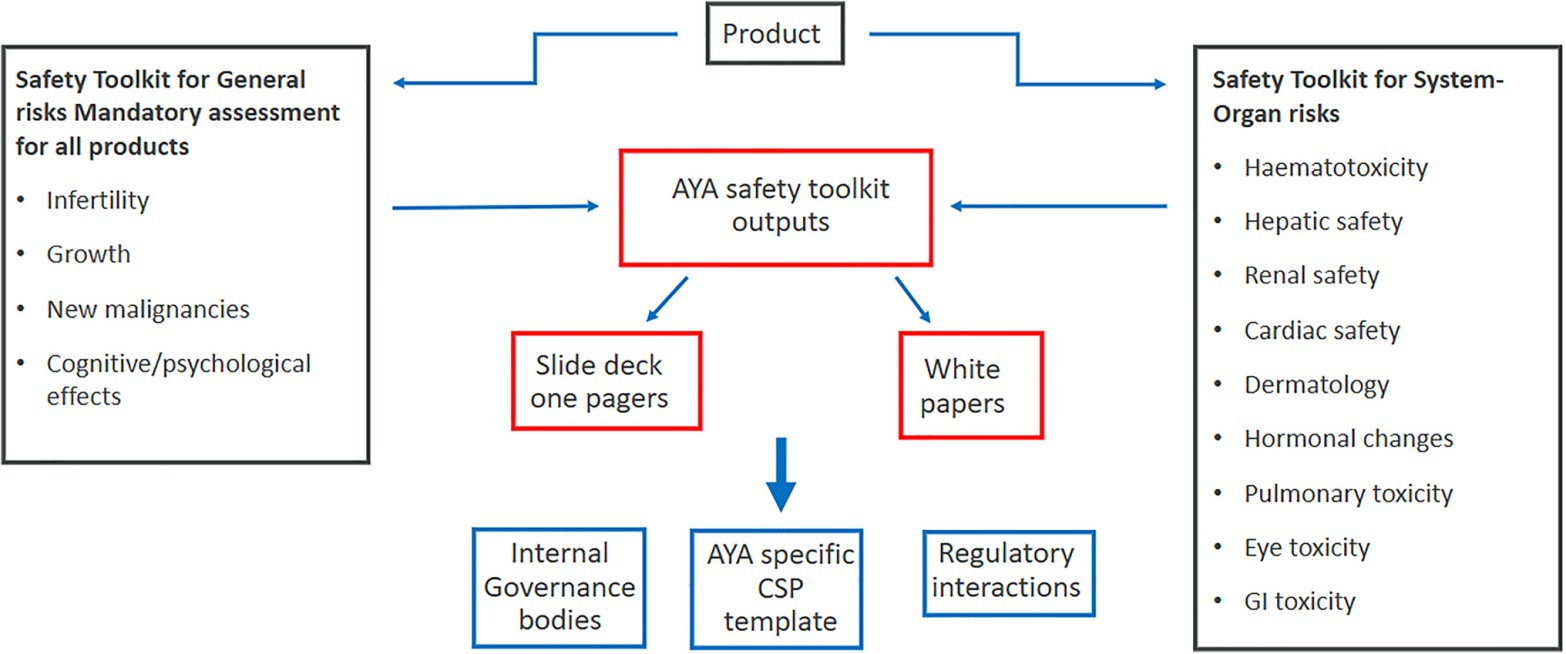
Decision Tree for Identifying Protocol Changes in Oncology Clinical Trials Including AYA Patients. This decision tree illustrates the process for determining general and system-organ specific risks that should be taken into consideration when assessing the benefit/risk of including AYA subjects in oncology clinical trials. This decision tree outlines key risks for consideration and based on those risks and their relevance to the product and its’ risks, helps determine if a protocol change is needed.

In instances where there are safety risks that are different for the AYA population compared to pediatric or adult patients, the toolkit will assist in identification of the differences. Some of these concerns are considered general safety risks, and they are relevant for all studies and products. General safety risks include infertility/fertility preservation, endocrine function/growth impact, risk for new malignancies and neurologic/psychological effects [11]. In addition to these general risks, the AYA toolkit guides the safety teams through consideration of other common or relevant system-organ risks such as toxicity impacting the blood, liver, heart, skin, lungs, eyes, or gastrointestinal tract (See Fig. 1). All system specific risks are assessed in parallel with consideration of the drug mode of action.

This paper describes the methodology for a decision tree on whether to include additional amendments to the adult protocols based on the safety principles described above.

## Methods

A series of safety topics, based on system organ classes used in pharmacovigilance, in tandem with identified general risks based on AYA literature, were selected for review, to determine whether there would be an impact on an adult oncology protocol (yes or no) and what additional AYA related wording is needed in the protocol (Fig. 1). The general risks are considerations for all AYA subjects regardless of the mechanism of the investigational product, whilst organ specific risks are related to the specific product specific mode of action and class effect.

For each of these safety topics the following were considered: organ-specific growth and maturation and reserve capacities, AYA-specific normal laboratory ranges, relevant common and/or important toxicities of anti-cancer therapies on the target organ, unique toxicities in AYA individuals, potential long-term consequences for AYA population, AYA-specific tolerability, common treatments of organ toxicity to cancer treatment and appropriateness for use in AYA individuals, toxicity management guidelines and AYA-specific safety monitoring requirements.

These considerations were assessed with literature searches, review of pediatric study protocols, review of regulatory guidelines and consultation of both pediatric and organ subject matter experts (internal and external). Explanations of the research findings were detailed in white papers prepared for each risk and AYA-specific additions to clinical protocol templates were developed.

## Results

The following tables outline the systematic assessment methodology outline for use of the toolkit, assessing the General Risks and the organ specific risks (Tables 1, 2, respectively). Each risk has simple binary, yes/ no decision options to indicate the necessary impacts on the protocol, allowing a simple but efficient visualization of the magnitude of necessary protocol changes.

**Table 1:**
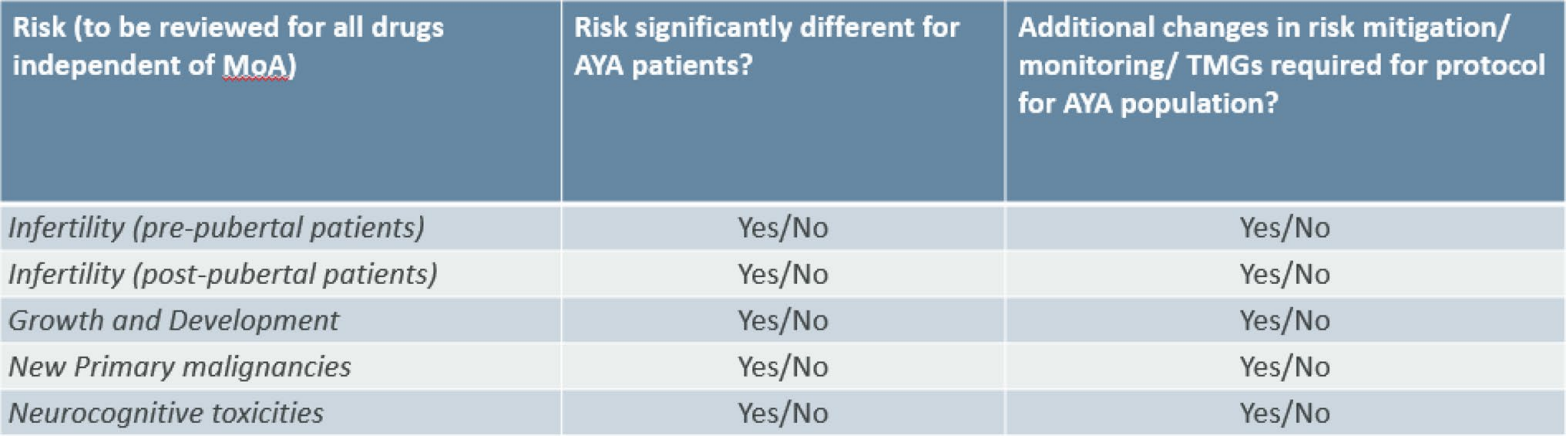
Assessment of general risks.

**Table 2:**
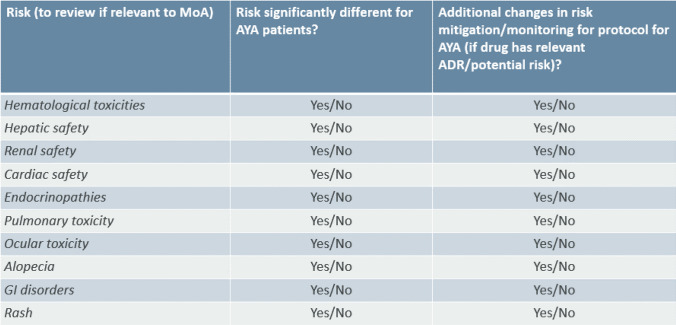
Assessment of organ-specific risks.

### Assessment of General Risk

The assessment of General Risks is mandatory for all products that are considered for the AYA population, however their impact on the protocol may differ, depending on the mode of action, patient population or the disease concerned.

In our resultant toolkit for General Risks, the infertility/fertility preservation topic was split into pre-pubertal and post-pubertal patients. As the infertility risk for AYA patients that have passed puberty should be no different to the risk in adult patients, we do not consider protocol changes should be required for most medications in a post-pubescent patient. However, the risk could be significantly different for pre-pubertal patients and considered relevant to update the protocol. Fertility preservation options are currently experimental in boys and more data is needed on the efficacy of the ovarian tissue cryopreservation method currently used in girls [12]. If AYA are to be included after a risk/benefit assessment per each medication, the toolkit commonly recommends the addition of Tanner stage monitoring during physical exam visits as a part of the protocol standard schedule of assessments.

The risk of impact on growth and development may be significantly different for AYA patients compared to adults and a protocol update will be needed in most cases. AYA individuals in the age range of 12–18 years old, developmentally, are still growing, with greatest increases in bone mass occurring during teenage years [13, 14]. If the decision is made to include AYA patients who are still growing in a clinical trial, changes to adult protocols include using growth charts to monitor growth at baseline and throughout study, and wrist x-ray screening to monitor growth plate fusion.

Because the risk of new primary malignancies may exist in AYA patients, recommendations for long-term follow up and cancer screening after study completion should be followed. As the AYA population would be expected to live longer than the adult population, then the overall risk may be higher for new primary malignancies. Our conclusion is that there may be no impact on the protocol, however patients should be informed if a future malignancy risk is known to exist, based on the properties of the study treatment.

Neurocognitive dysfunction in AYA patients may differ from adult patients due to the impact on neurologic function during a key phase of psychosocial growth and development [15–17]. AYA patients may also demonstrate a decline in cognition associated with risk factors of neuroinflammation, prior to initiation of systemic cancer therapy [17]. Age-appropriate psychosocial support is universally recommended, even in the absence of neurologic effect of medications. Cognitive impairment resulting from the effects of medications is documented for standard chemotherapy agents and should also be carefully evaluated for investigational agents. However, as a general risk, specific changes in the protocol should not be needed, unless the mode of action of the drug indicates a product specific risk that needs close neurologic monitoring.

### Assessment of Organ -Specific Risks

As part of the toolkit, system-organ safety risks were also reviewed. Teams are advised to go through a checklist and indicate whether the product specific risk is different for the AYA patients and whether protocol amendment is needed. The importance of each risk and whether significant changes are required to the protocol is dependent on the specific mode of action of the investigational medicinal product. The safety toolkit also describes where it is appropriate to use different laboratory reference ranges for AYA and adults for each system organ class. Detailed discussion of the recommendations for each of the individual topics is out of the scope of this paper and will be discussed in a future publication.

### Implications of the Results and Further Benefits of the Approach for Company Governance and Investment Decisions- the RAG Rating

**Figure 2:**
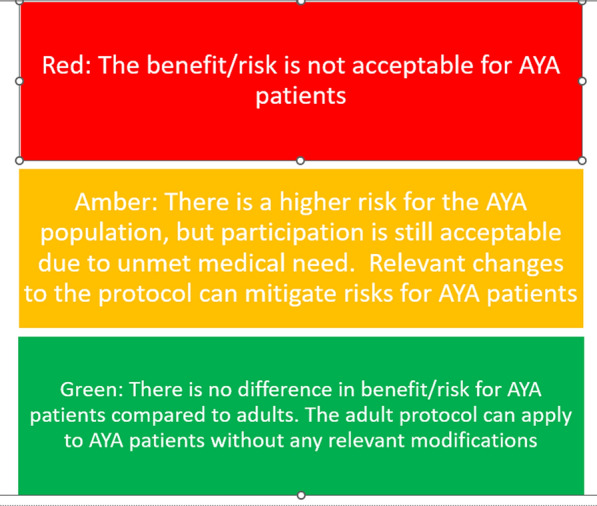
The Safety RAG rating score.

Once product teams use the safety toolkit, they are enabled to make a detailed assessment of the benefit-risk ratio of the medicinal product for AYA patients and anticipate the feasibility of including AYA patients in the study, including allocation of site personnel resources and assessment of cost elements. This will help with the development decision of whether it is safe to lower the age of inclusion in a particular clinical trial. A key deliverable from the evaluation of the risks is the generation of a safety Red-Amber-Green (RAG) rating for each study. The RAG rating scale is intended to summarize the scope and severity of any specific treatment-related safety concerns (Fig. 2). A red rating would indicate an unacceptable benefit/risk profile for AYA with the proposed treatment plan. An amber rating signifies there may be increased safety risk compared to adults, however the benefits of an unmet medical need balance the risk. For an amber rating, protocol modifications can be made to mitigate risk. A green rating indicates no expected additional risk and/or no expected relevant protocol modifications for AYA patients to safely proceed in the study. Any protocol modifications beyond adjustments for age-appropriate normal values in laboratory or diagnostic values, is considered a relevant modification.

### Case Example

During development of this toolkit the feasibility and application of using the toolkit for review was tested for a study investigating a medicinal product for women with locally advanced cancer. The team used the draft AYA toolkit to carefully evaluate treatment-related risks. A systematic review was conducted of each organ class and implications for mitigation of AYA-specific support was determined. (Table 4) After a thorough review of the safety data, a RAG rating of amber was assigned, and additional or modified monitoring and mitigation procedures was added to the clinical study protocol for AYA patients (Table 5).

In a worked risk evaluation case example, to understand the utility of the safety toolkit, a pharmacovigilance team reviewed an investigational agent and developed an assessment of the benefit/risk balance for inclusion of AYA for the product. (Tables 3, 4, 5) This is an example if a medication was noted to have potential adverse effect in adult patients on the cardiac system (e.g. myocardial infarction, congestive heart failure, tachycardia) and the pulmonary system (e.g. pulmonary fibrosis, pneumonitis, pulmonary artery hypertension).

**Table 3:**
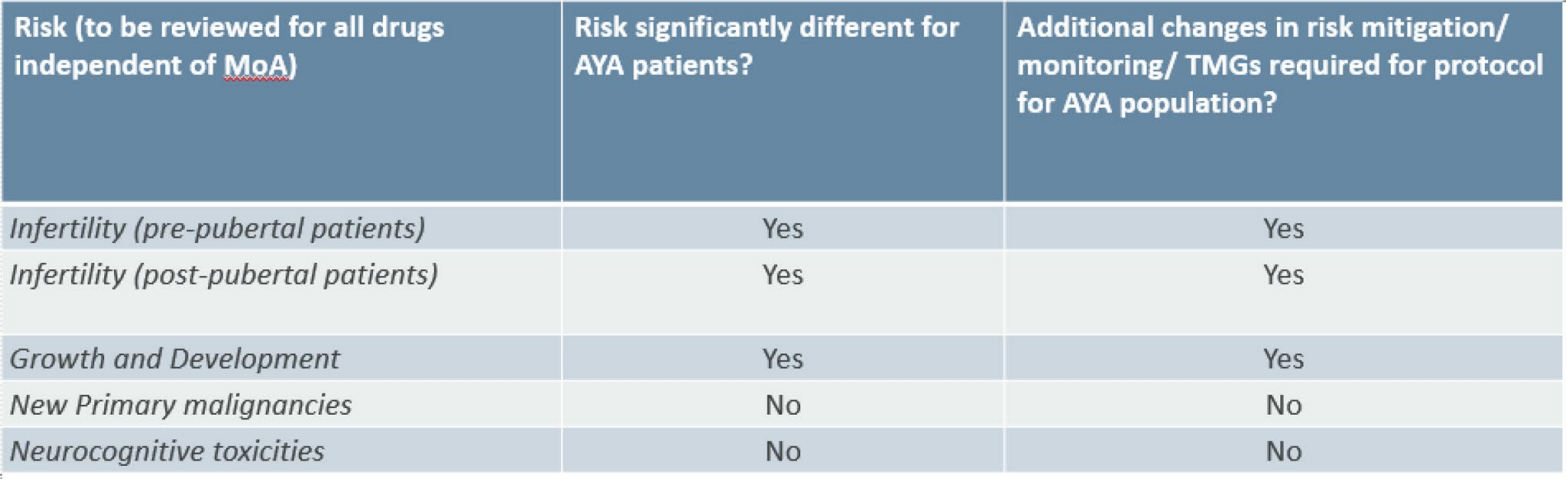
Risk evaluation of general risks.

**Table 4:**
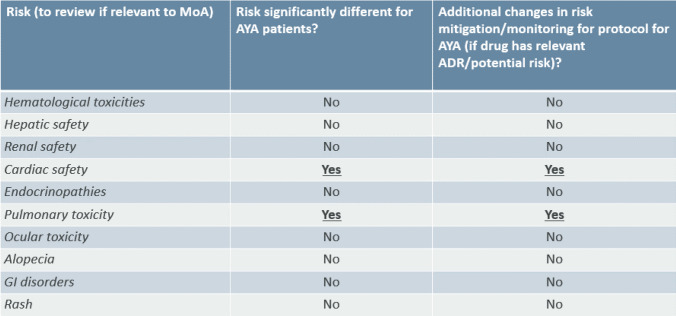
Risk evaluation of organ specific risks.

**Table 5:**
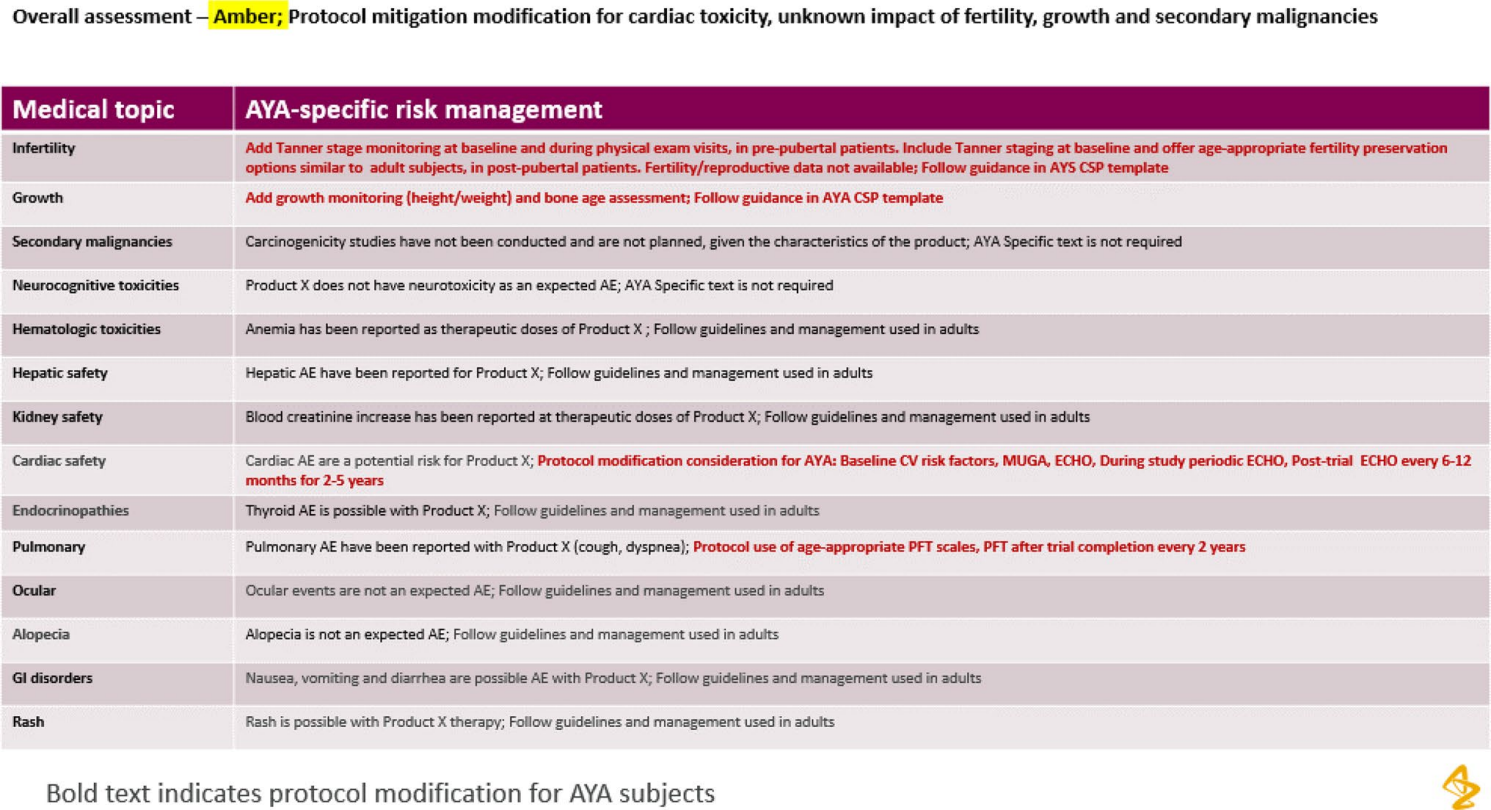
Risk evaluation and changes to protocol.

For general risks, the protocol added physical assessments for growth and sexual maturity, based on potential risks to endocrine function. Based on pre-clinical and clinical data, there is no expected impact on fertility, however the impact is not fully understood at this time. This created missing information in terms of fertility risk. Organ specific potential risks were identified for pulmonary and cardiac systems, and additional long-term tests for cardiac and pulmonary function were recommended, using age-appropriate scales.

The overall assessment for the worked case example, was an amber rating, indicating to proceed with increased monitoring, and conveying the message that the risk/benefit balance was encouraging for inclusion of AYA in clinical trials. The rationale for generation of an amber rating was the investigational product safety profile had some missing information in terms of fertility and also potential long-term adverse effects for cardiac and pulmonary systems, which may apply to many early phase study products. There were some monitoring adjustments to the protocol to monitor for the potential adverse effects, based on the known safety profile. However, in the setting of a cancer diagnosis with unmet need, it was agreed that the possible clinical benefits outweigh the anticipated risks, therefore it was advisable to continue the development program in AYA patients.

The protocol monitoring adjustments are intended to identify and mitigate impact of treatment on organ specific organ systems to adjust for age-appropriate identification of existing organ dysfunction and to put a framework in place to predict and monitor for long-term toxicities in subjects with prolonged survival.

## Discussion

It has been well documented that AYAs with cancer have less satisfactory clinical outcomes and have made slower progress in cancer advancements compared to pediatric and older adults. One of the identified key challenges and barriers to improving AYA cancer results is the lack of clinical trials available to this population [10]. Lack of safety and long-term safety data, in particular, for the AYA population has been identified as a key area that reduces inclusion of AYA patients in adult oncology clinical trials. We developed a simple safety toolkit to give clinical trial sponsors a comprehensive resource to overcome this challenge. The manner that the toolkit can assist in optimizing care is to improve access to early phase, new modalities of cancer drugs, decrease arbitrary age eligibility criteria, if no biologic reason for exclusion exists and increase physician awareness of trials to offer AYA patients.

In this article we describe a safety toolkit that enables pharmacovigilance and clinical development professionals to methodically evaluate how to safely include AYA individuals in adult oncology trials and impact investment decisions related to development of new therapies in AYA patients through the use of the RAG rating scale. The toolkit guides teams systematically through assessment of key, general risks as well as detailed organ-specific risks, that may be applicable depending on the pharmacodynamic characteristics and safety profile of the investigational product. Also, accompanying AYA protocol template language provides a consistent approach to include risk and mitigation strategies across clinical studies.

This method of detailed and biology-based review will help product teams to overcome concerns regarding management of safety risks in AYA patients. Historically the default position was not to lower the age of inclusion to AYA due to lack of safety data. Missing safety data, including long-term safety data should no longer considered as a default “red flag” without careful evaluation.

The utility of the toolkit was tested during set up of a new study investigating a new medicinal oncology product. After review of the general and organ-specific safety topics and consideration of the drug mechanism of action and safety profile, AYA-specific language was added to include growth and development monitoring during scheduled growth evaluation during physical exams and age adjusted modification of organ function inclusion criteria. This protocol has since been approved by regulators and has begun successful recruitment of study subjects.

One of the limitations of this toolkit is that there has been limited regulatory feedback as the toolkit has only recently been implemented. The utility of the toolkit will be continually assessed and refined further based on additional regulatory feedback, for future protocols. Additionally, the scope of this toolkit is for the use during and immediately after the clinical trial; survivorship guidelines (such as those established by ESMO and NCCN) should be referenced in order to address long-term monitoring.

## Conclusion

In summary this AYA oncology safety toolkit, including AYA-specific protocol template language, will help to increase the proportion of AYA patients enrolled on clinical trials and hopefully lead to better understanding of this patient population and improved survival.

## Data Availability

No datasets were generated or analysed during the current study.
